# Rheological, electrochemical, surface, DFT and molecular dynamics simulation studies on the anticorrosive properties of new epoxy monomer compound for steel in 1 M HCl solution†

**DOI:** 10.1039/c8ra09446b

**Published:** 2019-02-05

**Authors:** Omar Dagdag, Ahmed El Harfi, Omar Cherkaoui, Zaki Safi, Nuha Wazzan, Lei Guo, E. D. Akpan, Chandrabhan Verma, E. E. Ebenso, Ramzi T. T. Jalgham

**Affiliations:** Laboratory of Agroresources, Polymers and Process Engineering (LAPPE), Department of Chemistry, Faculty of Science, Ibn Tofail University BP 133 14000 Kenitra Morocco; Higher School of Textile and Clothing Industries, Laboratory REMTEX BP 7731, Oulfa Casablanca Morocco; AlAzhar University-Gaza, Chemistry Department, Faculty of Science P. O. Box 1277 Gaza Palestine; King Abdulaziz University, Chemistry Department, Faculty of Science P. O. Box 42805 Jeddah 21589 Saudi Arabia; School of Materials and Chemical Engineering, Tongren University Tongren 554300 China; Department of Chemistry, Faculty of Natural and Agricultural Sciences, School of Chemical and Physical Sciences, North-West University Private Bag X2046 Mmabatho 2735 South Africa chandraverma.rs.apc@itbhu.ac.in Eno.Ebenso@nwu.ac.za; Material Science Innovation & Modelling (MaSIM) Research Focus Area, Faculty of Natural and Agricultural Sciences, North-West University Private Bag X2046 Mmabatho 2735 South Africa; Department of Oil and Gas, Faculty of Engineering, Bani Walid University Bani Walid Libya

## Abstract

A new epoxy monomer, namely, tetraglycidyl-1,2-aminobenzamide (ER), was synthesized by condensation of the amines with epichlorohydrin in a basic medium. The obtained epoxy monomer was characterized by FT-IR and ^1^H NMR spectroscopy. Rheological properties of this monomer were determined using an advanced rheometer. Subsequently, the synthesized ER monomer was investigated as corrosion inhibitor for carbon steel in 1 M HCl solution. The adsorption properties of ER were analyzed by electrochemical, surface investigation and theoretical computational studies using DFT and molecular dynamics (MD). Results showed a high dependence of the viscosity of ER on temperature and concentration, and also, that ER has better inhibition performance. A good agreement between the results derived from computational (MD and DFT) and experimental methods was observed. The thermodynamic parameters, along with the kinetic parameters, showed that the adsorption of ER molecules onto carbon steel surface obeyed the Langmuir isotherm model, and the adsorption at metal–electrolyte interfaces involved both chemical and physical adsorption, but predominantly chemisorption mechanism.

## Abstract 2

A new epoxy monomer, namely, tetraglycidyl-1,2-aminobenzamide (ER), was synthesized by condensation of the amines with epichlorohydrin in a basic medium.

## Graphical abstract

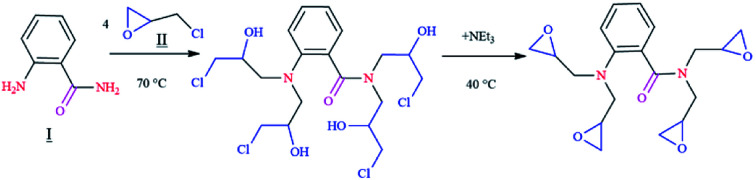

## 1 Introduction

Steel alloys are widely applied in numerous industries, such as the petroleum and construction industries, due to their low cost, availability and outstanding mechanical and physical properties. However, carbon steel is susceptible to corrosion in an environment containing chloride ions, especially acid media, which are frequently utilized in engineering procedures, including oil well acidification, acid curing and industrial acid cleaning.^1–3^ The use of corrosion inhibitors, mainly organic heterocyclic compounds, has been reported to be one of the most operational and functional approaches against corrosion, as these can easily interact and adsorb on metal surfaces *via* electron-rich centers constituted mainly of heteroatoms (P, O, S, N), π-electrons of multiple (double and triple) bonds and phenyl rings.^4,5^ Many of these organic heterocyclic compounds are obtained efficiently using easy and cost-effective approaches, and they show relatively high inhibition effectiveness.^6^

Aromatic resins, such as those based on bisphenol A-epichlorohydrin condensates, are a widely known group of epoxy monomers. The synthetic procedures and characterization of these epoxy monomers have been discussed in detail in the literature.^7–9^ The epoxy monomers are widely used because of their excellent adhesion performance.^10^ In our laboratory, we have successfully synthesized some aromatic epoxy monomers, employing reagents containing at least two mobile hydrogens of diamine type and alcohols (mostly with low molecular weights) to react with epichlorohydrin.

In this study, we synthesized the epoxy monomer, namely, tetraglycidyl-1,2-aminobenzamide (ER), and characterized it by FT-IR and ^1^H NMR spectroscopy. The rheological property of the epoxy monomer was determined by the HAAKE MARS rheometer. Moreover, this epoxy monomer contains several electron-rich centers in the form of heteroatoms and π-electrons, through which they can adsorb effectively and thereafter inhibit corrosion. To this end, we report the inhibition effect of the epoxy monomer ER against carbon steel corrosion in 1 M HCl acidic medium using experimental (electrochemical) and computational techniques.

## 2 Experimental

### 2.1. Materials and chemicals

**Fig. 1:**
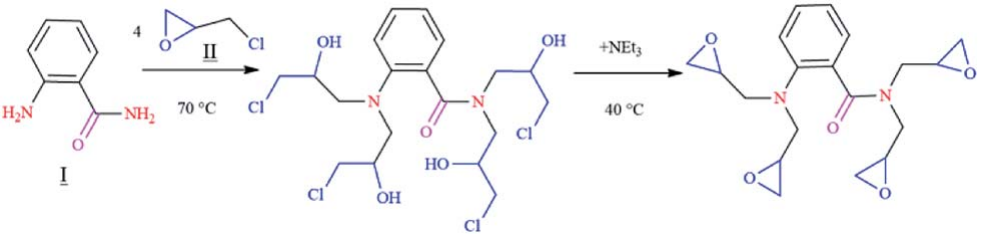
A representative scheme showing the synthesis of tetraglycidyl-1,2-aminobenzamide (ER).

The chemicals and materials used in this work, such as 1,2-aminobenzamide (98%), epichlorohydrin (99%) and triethylamine (≥99.5%) were purchased from Aldrich Chemical Company. Composition of carbon steel is given in Table SI 1.† The corrosive solution of 1 M HCl was prepared by diluting the appropriate amount of concentrated HCl (37%) with distilled water. The range of concentration for the tested inhibitor was from 10^−3^ to 10^−6^ mol L^−1^. Synthesis of the epoxy monomer ER was carried out as shown in Fig. 1 by reacting 1,2-aminobenzamide (I) with epichlorohydrin (II) in the presence of triethylamine, according to a procedure reported in the literature.^7–9^

#### 2.1.1. Synthesis of tetraglycidyl-1,2-aminobenzamide (ER)

The compound ER was synthesized by the reaction of 2.72 g of 1,2-aminobenzamide (2 × 10^−2^ mol) and 5 ml of epichlorohydrin in ethanol. The mixture was heated to 70 °C and kept for about 4 h. Then, it was cooled to 40 °C, and 6.5 ml of triethylamine was added dropwise for 3 h. Finally, the system was allowed to cool to room temperature. The mixture was then dried in vacuum. The structure of the epoxy monomer was confirmed by ^1^H NMR (Fig. SI 1†) and FT-IR spectroscopic characterization techniques. The spectroscopic data are presented in Table SI 2.† Progress of the reaction was determined using thin-layer chromatography, and purity of the product was determined using a single spot on the TLC plate. The synthetic yield of ER was 92%.

### 2.2. Rheological measurements

The rheological properties of the epoxy monomer ER were determined with a HAAKE MARS rheometer (Thermo-Scientific) with the cone-plate geometry (the diameter of plateau is 35 mm and cone angle 1°). Shear viscosity (*η*) was obtained by employing an increasing shear stress ramp at a constant stress rate. All the measurements were performed in duplicate.

### 2.3. Electrochemical measurements

The effect of ER on the corrosive dissolution of carbon steel was studied using electrochemical techniques. Before doing the electrochemical measurements, the carbon steel specimens were abraded using different grades (180 to 1000) of emery paper, washed with distilled water and ethyl alcohol, and finally, degreased with acetone. For electrochemical measurements, a three-electrode setup was used, consisting of a reference electrode constituted by saturated calomel electrode (SCE), a counter electrode of Pt ring and working electrode (WE) of carbon steel specimen. All electrochemical measurements were performed after 30 min immersion time. For electrochemical measurements, the exposed WE area was 1 cm^2^. The electrochemical behavior of ER was studied using a potentiostat instrument (BioLogic SP-200) as described in our earlier reports.^9,10^

The electrochemical impedance spectroscopy (EIS) measurements were performed over a frequency range of 100 kHz to 10 mHz, using 10 mV amplitude. The Nyquist plots derived in the absence and presence of ER were fitted in the suitable equivalent circuit (described later) in order to obtain some useful parameters, including the charge transfer resistance (*R*_ct_). Inhibition efficiency *η*_EIS_% is calculated from charge transfer resistance *R*_ct_ of ER, according to eqn (1):1
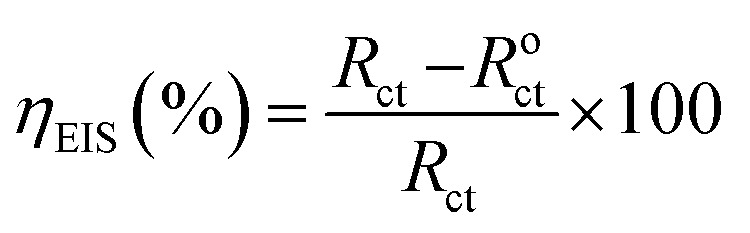
where *R*^o^_ct_ and *R*_ct_ represents the charge transfer resistance without and with epoxy monomer ER, respectively.

Results of the EIS study were supported by potentiodynamic polarization (PDP) measurement. The PDP curves were measured at a scan rate of 1 mV s^−1^, from −800 to 0 mV *versus* SCE. Anodic and cathodic Tafel curves for carbon steel dissolution with and without ER (in 1 M HCl) were recorded. Extrapolation of the linear segments of the anodic and cathodic curves gives the useful parameters, including anodic and cathodic Tafel slopes, corrosion potential (*E*_corr_), and corrosion current density (*i*_corr_). The corrosion current density (*i*_corr_) was used to calculate *η*_PDP_ (%) of ER, given by eqn (2):2
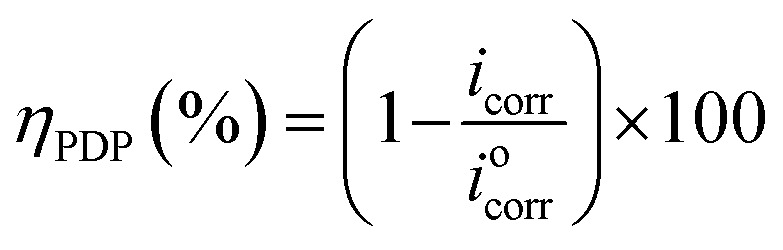
where *i*^0^_corr_ and *i*_corr_ represent corrosion current density obtained without and with ER, respectively.

### 2.4. Scanning electron microscopy (SEM)

The SEM study was conducted using the S3000H instrument (Hitachi) at the accelerating voltage of 20 kV. For SEM study, the carbon steel coupon was allowed to corrode in 1 M HCl solution without and with 10^−3^ M ER; thereafter, the surfaces of the coupons were examined for their morphological changes.

### 2.5. Computational details

#### 2.5.1. Quantum chemical calculations

In this research, the molecular sketches of the new epoxy compound and its protonated form were drawn using the Gauss View 53.0.^11^ Standard Gaussian 09 package^12^ was used to carry out all the quantum calculations. Geometry calculations of the new epoxy compound in both gas phase and aqueous medium were performed at the B3LYP/6-311+(d,p) level of theory.^13–16^ Solution study (aqueous medium, DC = 78.5) was performed using self-consistent reaction field (SCRF) theory with Tomasi's polarized continuum model (PCM).^17,18^ Vibrational frequencies of the molecule were computed by using the abovementioned level of theory, and no imaginary frequency existed, signifying negligible energy structures. According to Koopman's theorem,^19^ the *E*_HOMO_ and *E*_LUMO_ energies were used to calculate the global quantum chemical indices using eqn (3)–(7):^20–23^

3 The ionization potential: *I* = −*E*_HOMO_

4 The electron affinity: *A* = −*E*_LUMO_

5 The energy gap: Δ*E* = *E*_LUMO_ − *E*_HOMO_

6
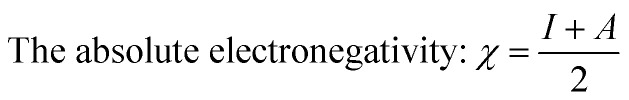
7
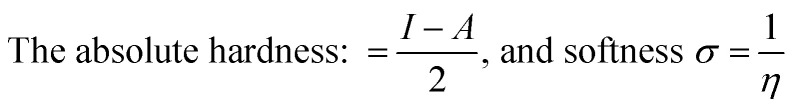


It is worth mentioning that both the stability and reactivity of a molecule can be measured by estimating the absolute hardness of a molecule.^24–26^ Generally, molecules with a large energy gap are known as hard molecules, whereas molecules possessing a small energy gap are referred to as soft molecules.^27,28^

The electrophilicity index (*ω*), which is a measure of the electrophilic power of an inhibitor, has been estimated using the following expression:8
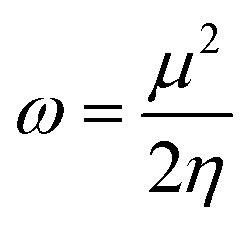
while the physical inverse of electrophilicity is known as the nucleophilicity (*σ* = 1/*ω*).

The calculated values *χ* and *η* of the inhibitors can be used to estimate the fraction of electrons transferred, Δ*N* (eqn (9)), which could be used to evaluate the ability of the inhibitor molecule to transfer electron density to the metal surface:^29–32^9
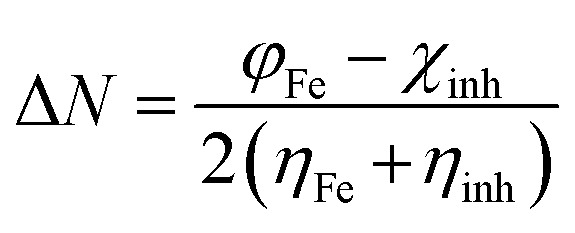


The theoretical value of the *η*_Fe_ is 0, and the values of the work function (*φ*) are 3.91, 4.82 and 3.88 eV for (1 0 0), (1 1 0) and (1 1 1) planes of Fe, respectively.^33,34^

The back-donation energy, Δ*E*_b-d,_ corresponds to the interaction existing between the inhibitor molecule and the metal surface and correlates with the hardness of the inhibitor, as shown in eqn (10):^35^10
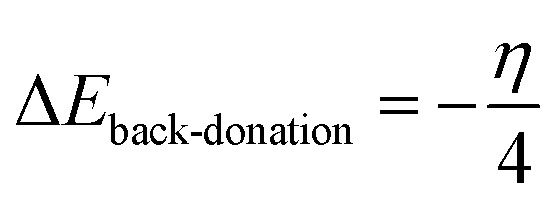


When *η* > 0 or Δ*E*_b-d_ < 0, the back-donation from the inhibitor to the metal surface is energetically favored.

Finally, the initial molecule–metal interaction energy (Δ*ψ*) index can be calculated using eqn (11):^29^11
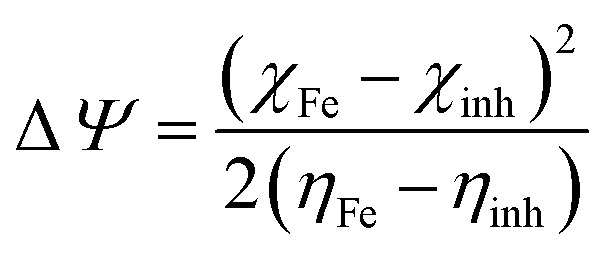
where a theoretical value of *χ*_Fe_ = 7 eV and *η*_Fe_ = 0 eV.^29^

#### 2.5.2. Local reactivity analysis (Fukui indices)

The local reactivity properties can be usefully compared using the Fukui functions.^36^ As described in earlier published literature, the Fukui functions are calculated as follows:^37^

12 *f*_k_^+^ = *q*_k_(*N* + 1) − *q*_k_(*N*) (for nucleophilic attack)

13 *f*_k_^−^ = *q*_k_(*N*) − *q*_k_(*N* − 1) (for nucleophilic attack)

In eqn (12) and (13), *q*_k_(*N*), *q*_k_(*N* + 1) and *q*_k_(N − 1) are the atomic charges of the systems with *N*, *N*+1, and *N* − 1 electrons, respectively.

#### 2.5.3. Molecular dynamics simulation

Forcite module of Materials Studio 8.0 program developed by BIOVIA Inc. was employed in the molecular dynamics (MD) simulation.^38–40^

## 3 Results and discussion

### 3.1. Mechanism of epoxide ring opening

Water, alcohols and many other reagents have exerted significant effects on epoxy resin ring opening. These reactions serve as basis for the production of glycols and/or the formation of epoxy glues.^41^ Under acidic conditions, nucleophilic addition is affected by steric effects that occur mainly by S_N_^2^ mechanism, as well as the stability of emerging carbocation, which occurs by S_N_^1^ mechanism. Hydrolysis of an epoxide ring in acid medium generates glycol, as shown in Fig. SI 2.†

### 3.2. Rheological properties

#### 3.2.1. Effect of concentration

**Fig. 2:**
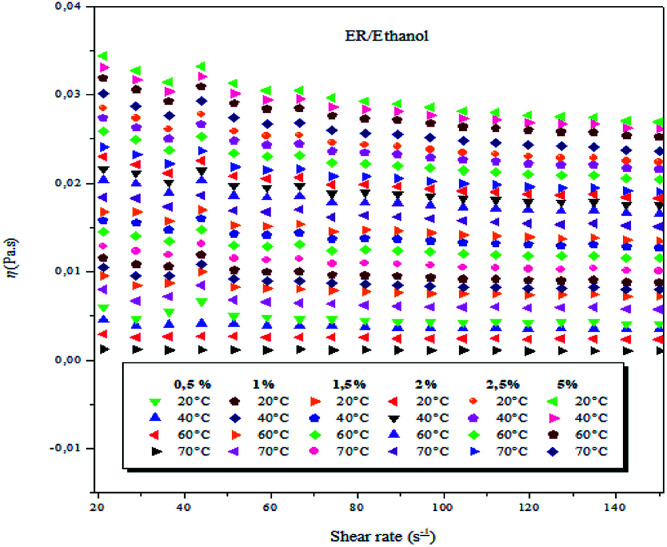
Viscosity as a function of shear rate of ER/ethanol solutions at 0.5, 1.0, 1.5, 2.0, 2.5 and 5 wt% (weight concentrations) and varying temperatures.

The viscosity of the epoxy monomer ER/ethanol was measured as a function of shear rate at various concentrations (0.5, 1, 1.5, 2, 2.5, 5 wt%) and at different temperatures, in the range of 20–70 °C, as shown in Fig. 2.

In the present study, at the different studied concentrations (0.5–5 wt%), the ER/ethanol acted as a non-Newtonian liquid, and the viscosity was dependent on the shear rate at temperatures between 20 and 70 °C, ranging from 0.01 to 150 s^−1^. A decrease in viscosity with a corresponding increase in the shear rate was observed, suggestive of the fact that the epoxy monomer ER/ethanol showed a non-Newtonian rheological behavior of pseudo-plastic type.^42^ The viscosity behaviors of epoxy monomer ER/ethanol as a function of shear rate depends on temperature and concentration, respectively. As anticipated, the viscosity of ER increases with increase in ER concentration, primarily resulting from the increase in the molecular interactions between the epoxy monomers (entanglements, see Fig. SI 3).†

#### 3.2.2. Effect of temperature

The effect of temperature on the viscosity of ER was also evaluated, and results are summarized in Fig. SI 4.†The results show that the viscosity decreases as the temperature increases, and it could be said that the rheological properties of ER/ethanol are highly temperature dependent. This could be possible because of the migration of segments of molecules or individual molecules (epoxy monomers) in jumps, from one place in a lattice to a vacant hole of the lattice. The overall area available in the lattice is defined as ‘‘hole concentration”. The relationship between the ln(*η*_0_) *vs.* 1/*T* for the epoxy monomer at different concentrations is shown in Fig. SI 5.† The data acquired on the flow of the molten epoxy monomer permits the valuation of other rheological parameters, such as flow activation energy. Using the observation that the viscosity changes with temperature and obeying the Arrhenius equation, we have calculated the activation energy (*E*_a_) of the resins using eqn (16):^43^14
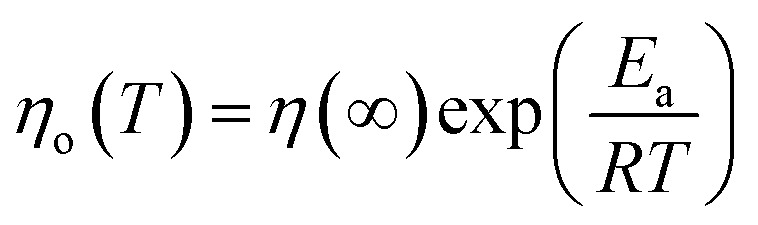
where *η*_0_ represents zero shear viscosity at temperature *T*, *η*(∞) is a constant, *E*_a_ is the activation energy of each concentration of ER/ethanol, *R* the gas constant and *T* represents temperature in K.

The activation energy for ER/ethanol varies between 0.60–11.90 kJ mol^−1^. The obtained results indicate the high activation energy for ER/ethanol. *E*_a_ expresses the difficulty of the epoxy monomer molecules’ movement from one position to another, which is controlled by the intermolecular forces, bulkiness and rigidity. The effect of molecular structure on the value of *E*_a_ has been reported by many authors.^42^

### 3.3. Electrochemical measurements

#### 3.3.1. Open circuit potential (OCP)

**Fig. 3:**
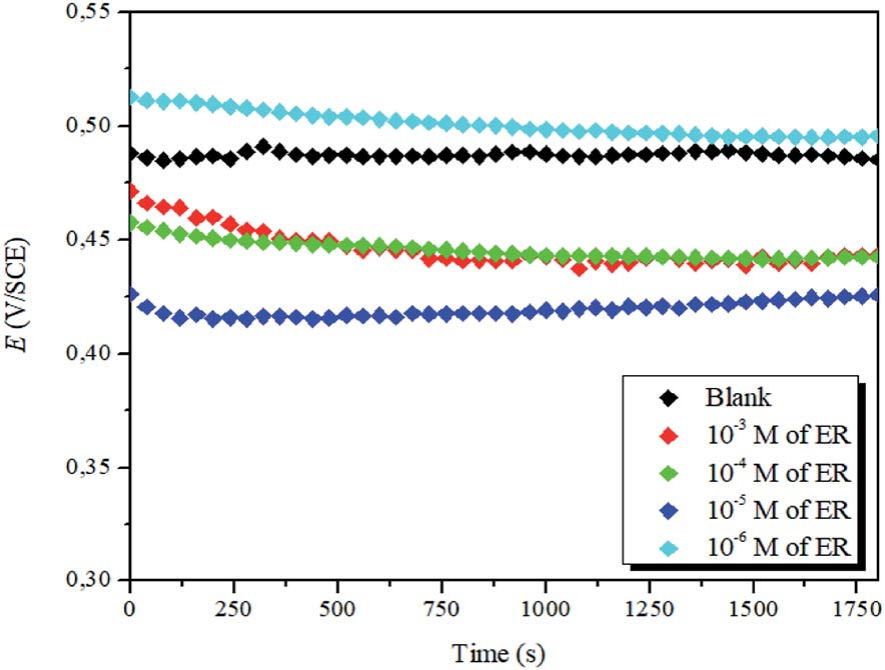
Evolution of OCP against time for the corrosion of steel in 1 M HCl with varying concentrations of ER at 298 K.

The plots of variation of OCP against time at different concentrations of ER are presented in Fig. 3.

It is evident that the addition of the inhibitor molecules brings a continuous shift in OCP (*i.e.*, *E*_corr_) to anodic potentials. Straight OCP *versus* time curves reveal that adsorption of the ER takes place after the dissolution of surface oxide (Fe_3_O_4_ and Fe_2_O_3_) layers.

#### 3.3.2. Potentiodynamic polarization (PDP)

**Fig. 4:**
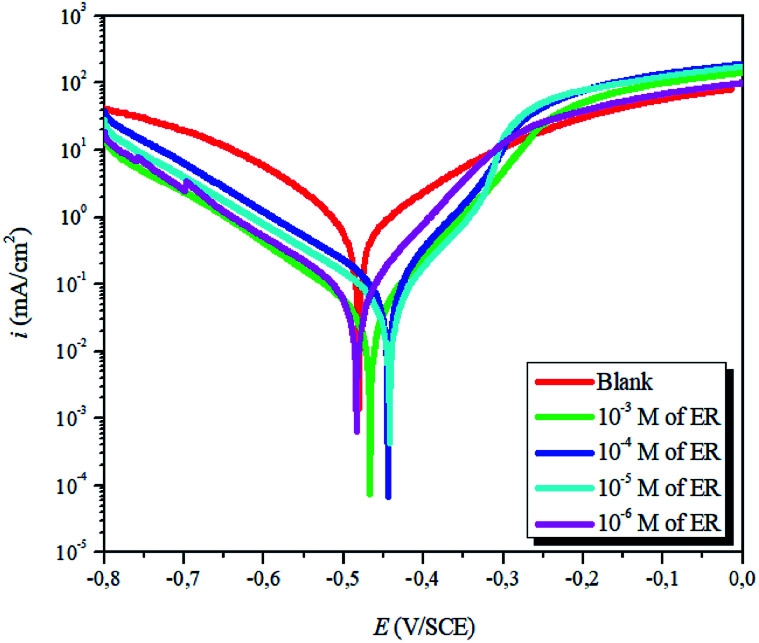
Potentiodynamic polarization plots for carbon steel corrosion in 1 M HCl solution without and with varying concentrations of ER.

The PDP plots for carbon steel in 1 M HCl solution without and with various concentrations of ER at 298 K are depicted in Fig. 4. The values of polarization parameters are as represented in Table 1.

**Table 1:** Tafel polarization parameters for carbon steel in 1 M HCl solution without and with varying concentrations of ER

Inh	*C* (M)	*E*_corr_ (mV)	*i*_*c*orr_ (μA cm^−2^)	*β*_a_ (mV dec^−1^)	−*β*_c_ (mV dec^−1^)	*η*_PDP_%	*θ*
Blank	—	−473.80	916.6	163.6	155	—	—
	10^−3^	−467.05	34.31	78.1	126	96.0	0.960
ER	10^−4^	−444.53	87.22	70.5	135	90.5	0.905
	10^−5^	−469.74	88.38	128	152	90.3	0.903
	10^−6^	−455.70	108	128	136	88.0	0.880

The results show that in the presence of ER, *i*_corr_ values decreased sharply. Correspondingly, the highest inhibition efficiency of 96% was obtained at 10^−3^ M concentration. This value suggests that ER acted as an efficient corrosion inhibitor against carbon steel corrosion in acidic solution. Furthermore, as presented in Fig. 4 and Table 1, the addition of ER molecules shifted the corrosion potential (*E*_corr_) slightly to a more positive direction and also shows changes in both the cathodic and anodic polarization branches. These results indicate that the added epoxy monomer ER acted as an anodic-type inhibitor.

#### 3.3.3. Electrochemical impedance spectroscopy (EIS) measurement

**Fig. 5:**
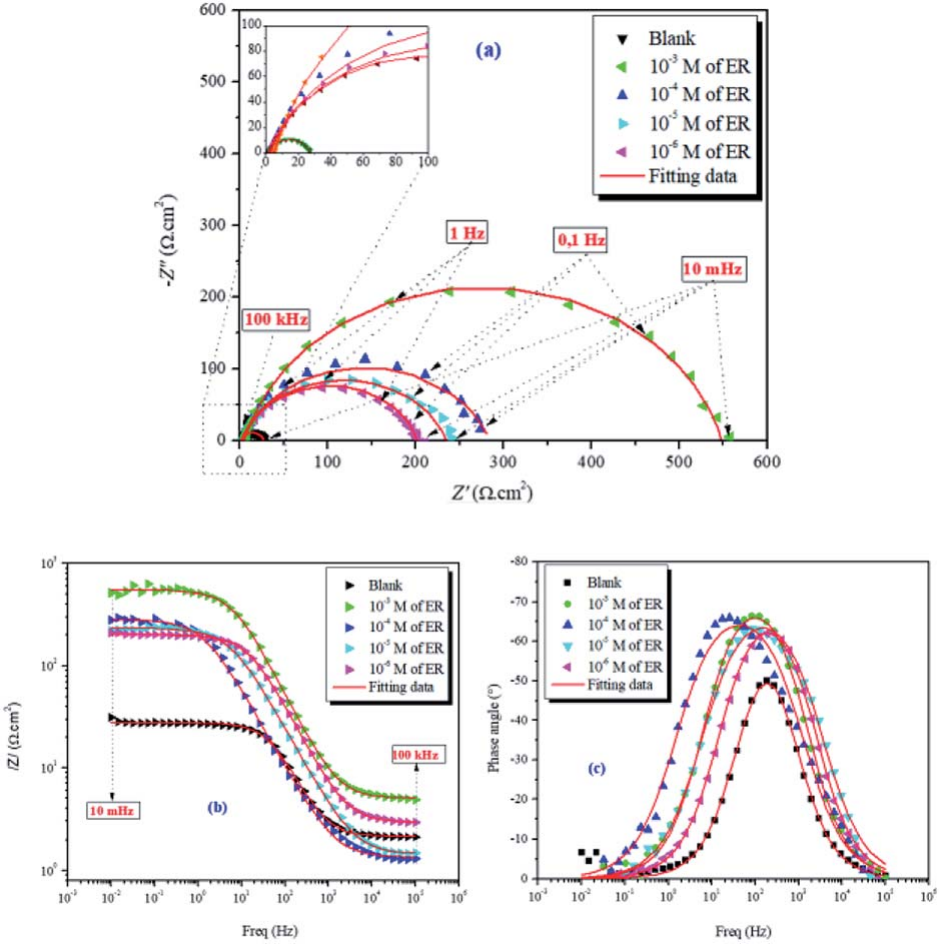
Nyquist (a) and Bode (b and c) diagrams for carbon steel in 1 M HCl solution without and with varying concentrations of ER.

Fig. 5(a–c) denotes the Nyquist diagrams and Bode plots without and with varying concentrations of ER.

The values of electrochemical parameters such as *R*_s_, *R*_ct_ and *C*_dl_ of ER are presented in Table 2. A careful look at Fig. 5a clearly reveals only one capacitive loop in the Nyquist plots, which could be attributed to a single charge transfer.^44^ Meanwhile, the size of these loops increases with the rise of ER concentration up to 10^−3^ mol L^−1^, which signifies that ER was adsorbed on the carbon steel surface, and the area exposed to the corrosive 1 M HCl solution was reduced. The electrochemical equivalent circuit used to model the experimental data is depicted in Fig. SI 6.† The *R*_s_ and *R*_ct_ in the circuit signify the resistance of the solution and charge transfer resistance, respectively. The constant phase element (CPE) replaces the double-layer capacitor to allow for accurate fitting of the experimental results.^45,46^Eqn (15) was used to calculate the impedance of CPE:15
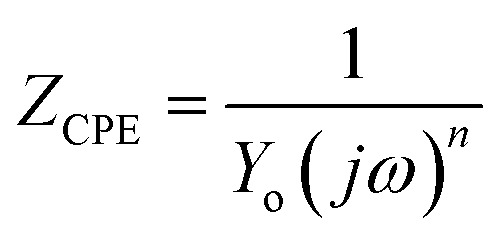


**Table 2:** Electrochemical impedance parameters for carbon steel in 1 M HCl solution without and with varying concentrations of ER at 298 K

Inh	*C* (M)	|*Z*|_0.01 Hz_ (Ω cm^2^)	*R*_s_ (Ω cm^2^)	*R*_ct_ (Ω cm^2^)	*C*_dl_ (mF cm^−2^)	*η*_EIS_%	*χ* ^2^
Blank	—	27	2.15	25	4.6	—	0.056
ER	10^−3^	538	5.04	543	0.08	95	0.148
	10^−4^	291	1.34	283	0.11	91	0.983
	10^−5^	229	1.43	234	0.24	90	0.189
	10^−6^	199	2.90	199	0.60	87	0.034

In the above equation, *n* denotes the phase shift. The value of *n* serves as a gauge for the measurement of CPE nature; for example, *n* = 1, *Y*_0_ = *C* indicate the capacitive behavior of the CPE; *n* = 0, *Y*_0_ = 1/*L* indicate the inductive behavior of the CPE; and *n* = 1/2, *Y*_0_ = *W* indicate the Warburg impedance. Additionally, high *n* value is related to high surface smoothness, and its low value is consistent with high surface roughness. In the present study, values of *n* are very close to unity, which indicates the CPE behaves as pseudo-capacitor.^47^

The magnitude of the double-layer capacitance (*C*_dl_) is derived as follows:

16 *C*_dl_ = *Y*_o_(*ω*_max_)^*n*−1^

where *ω*_max_ represents the frequency at which the imaginary impedance assumes the highest value (in rad s^−1^). The calculated values of *R*_ct_ and *C*_dl_, along with other EIS indices, are listed in Table 2. The decrease in *C*_dl_ values is related to the distribution of ionic charge at the charge metal interface/solution, which suggests that ER acted at the metal interface/solution. However, the increase in *R*_ct_ values can be ascribed to adsorption of inhibitors at the metal surface. The Bode impedance and phase angle plots are given in Fig. 5b and c. Increase in the impedance modulus (|*Z*|) towards lower frequency regions confirms the superior inhibition protection. The increased values of phase angle with varying concentrations of ER, as seen in the system, is an indication that the roughness of the metal surface has reduced significantly because of the formation of protective film by inhibitors.^48,49^

### 3.4. Morphological analysis

**Fig. 6:**
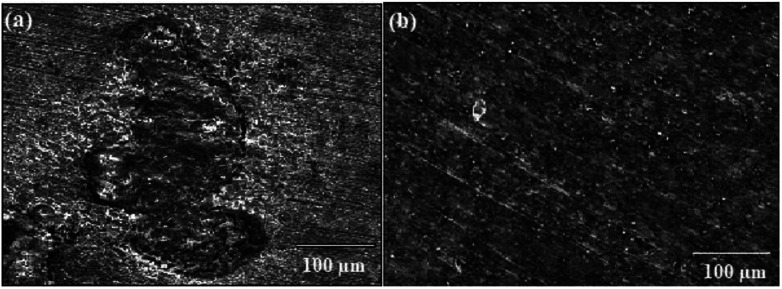
SEM micrographs of carbon steel after 12 h of immersion in 1 M HCl solution: (a) without inhibitor and (b) with 10^−3^ M ER.

The surface morphology of metal coupons after immersion in 1 M HCl solution without ER and with 10^−3^ M ER for 12 h were examined by SEM, and their micrographs are shown in Fig. 6. As seen in Fig. 6a, the SEM image revealed surface damage owing to the aggressive attack of 1 M HCl solution. On the other hand, the surface of the carbon steel immersed in the corrosive media for 12 h with 10^−3^ M ER shows a drastic reduction in surface damage (Fig. 6b). From the micrographs, it could be seen that there is adsorption of molecules of ER *via* the active sites of the metallic surface, resulting in a smoother surface (except for a few spots), signifying the protection of the metal from corrosion. Elemental composition of the metallic surface with and without ER was determined using EDX spectra, which are given in Fig. SI 11.†

### 3.5. Effect of temperature

To gain further understanding of the effect of temperature on the corrosion inhibition process, the inhibition ability of ER in 1 M HCl solution was investigated at 298, 308, 318 and 328 K. PDP measurements recorded at the respective temperatures are depicted in Fig. SI 7,† and the values for PDP parameters (*E*_corr_, *i*_corr_) and inhibition efficiencies are listed in Table SI 3.† A careful look at Table SI 3† reveals that increasing the temperature of the 10^−3^ M ER solution in 1 M HCl resulted in a corresponding increase in the values of *i*_corr_ and a decrease in protection efficiency (*η*%). This result shows that temperature affects the inhibition performance of ER in this temperature range (298–328 K), especially at lower concentration.

#### 3.5.1. Kinetic parameters of corrosion process activation

Generally, an increase in temperature causes significant decrease in the protection ability of ER. The influence of temperature can be best explained with the aid of Arrhenius' equation:^50,51^17
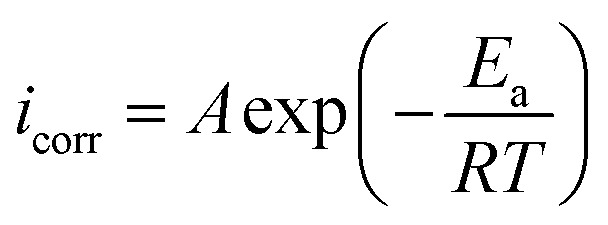


The Arrhenius plot obtained in this study is shown in Fig. SI 8.† Moreover, the Arrhenius equation can be converted to a transition-state equation, as shown in eqn (18):^44,52^18
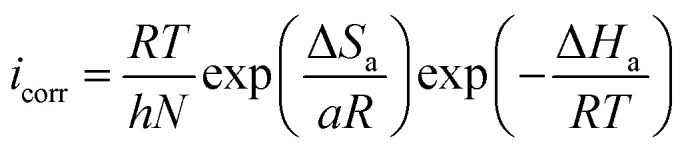


where, *T* is temperature, *N* is Avogadro's constant (6.022 × 10^23^ mol^−1^) and other symbols have their usual meaning. Several parameters, such as *E*_a_, Δ*H*_a_ and Δ*S*_a_, were calculated using Arrhenius and transition state plots (Fig. SI 9†) and are presented in Table SI 4.† The positive value of Δ*H*_a_ reveals the endothermic nature of metal-inhibitor interactions,^52^ whereas the increased value of Δ*S*_a_ suggests that replacement of the water molecules from ER resulted in a corresponding increase in the disorder (randomness) on the metallic surface.

### 3.6. Adsorption studies

Various adsorption isotherm models are frequently utilized to understand the mechanism of adsorption between the inhibitors and metal atoms at the surface. In the present study, Langmuir, Temkin, and Frumkin adsorption isotherms were employed to fit the results from PDP analysis of ER:^53^19
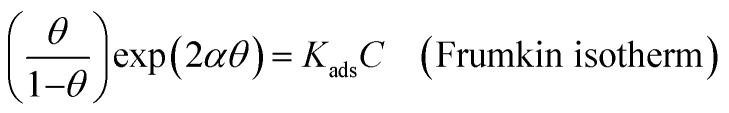
20
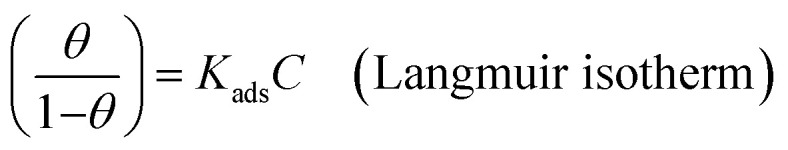

21 exp(−2*αθ*) = *K*_ads_*C* (Temkin isotherm)

where *θ* and *C*_inh_ are the surface coverage and concentration of ER, respectively. *K*_ads_ is the equilibrium constant of the adsorption–desorption (of ER) processes. A perfect linear regression coefficient (*R*^2^) was obtained when data obtained from PDP analysis was fitted into the linear Langmuir isotherm model, indicating that ER adsorption at the interface was consistent with the Langmuir adsorption isotherm model. As seen in Fig. SI 10,† the relationship between *C*_inh_ and *C*_inh_/*θ* gave straight lines, and *K*_ads_ was calculated from the intercept. The standard free energy of adsorption Δ*G*^o^_ads_ was obtained from eqn (22):^54^22
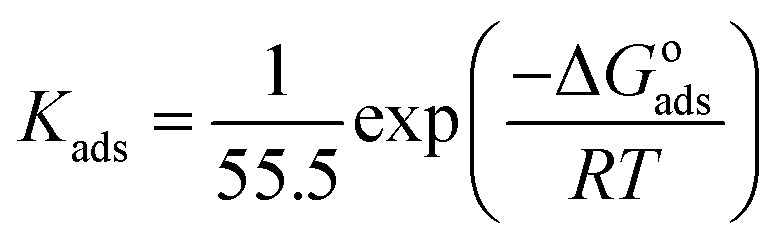
where *R* is the universal gas constant (8.314 J mol^−1^ K^−1^), and the value 55.55 is the water concentration in the solution (mol L^−1^). Literature reports confirm that a high value of *K*_ads_ and low value of Δ*G*^o^_ads_indicate a strong interaction and adsorption on the metal surface.^55^ The values of *K*_ads_ (0.345 M^−1^ × 10^6^) and Δ*G*^o^_ads_ (−42.12 kJ mol^−1^) obtained in this study indicate that ER exhibits a strong interaction with the carbon steel surface (chemisorption).^56^

### 3.7. Theoretical studies

#### 3.7.1. Quantum chemical calculation

**Fig. 7:**
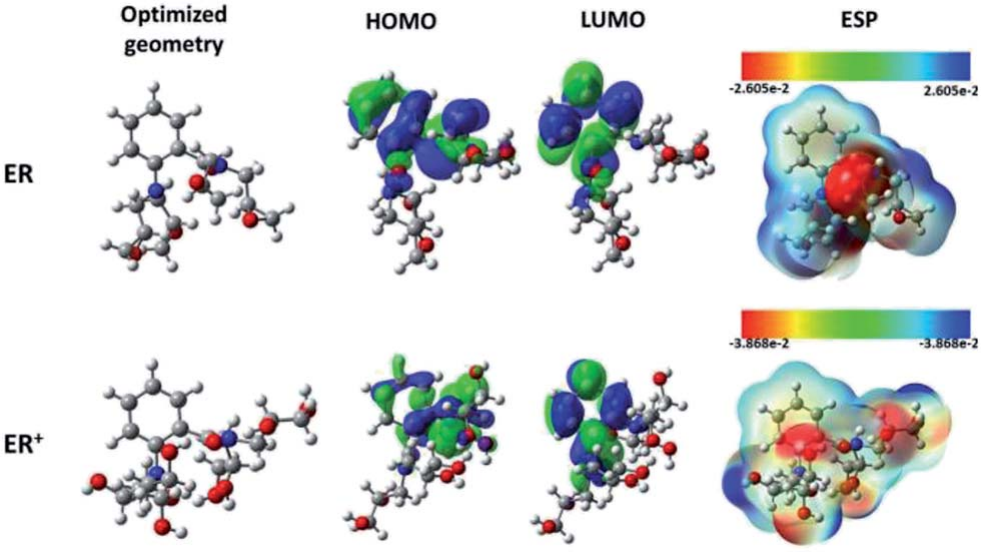
Chemical structures, optimized, HOMO, LUMO, and ESP images of neutral and protonated forms of the studied ER molecule using B3LYP functional and 6-31+G(d,p) basis set.

Recently, DFT has emerged as a powerful computation tool for describing the interactions occurring at the metal and inhibitor molecule interface.^57^Fig. 7 shows the B3LYP/6-311+G(d,p) fully optimized geometries and the HOMO, LUMO and molecular electrostatic potential (ESP) diagrams of the tested molecule and its protonated form. Analysis of the HOMO and the LUMO is very important to understanding the reactive sites of the molecules.

On the other hand, the electronic parameters can be used to study the reactivity of the tested molecule. Singh and co-workers^58^ showed that increasing HOMO energy leads to increase in the electron donation tendency of a compound. In contrast, decrease in the LUMO energy is a good indication of electron acceptance ability. Fig. 7 shows that the distribution of the HOMO is concentrated in the middle of the molecular structure of the tested molecule and its protonated form. The electronic distribution is mainly located on the aromatic benzene ring, the carbonyl group and on the nitrogen atoms, with less activity around the epoxy groups. It is also obvious that both the inhibitor and its protonated form have similar HOMO distribution. Interestingly, the LUMO electron densities of the tested molecule and its protonated form moved toward the non-reactive parts in HOMO distributions.

Rahmani and co-workers^59^ suggested that electron-rich and -deficient centers can be identified with the aid of molecular electrostatic potential (ESP). The ESP derived for ER is presented in Fig. 7. The values of the electrostatic potential have been used to evaluate the ESP maps. The most negative regions of the ESP have been given the red color, while blue stands for the regions of the most positive ESP, and green for zero ESP.^60^ Therefore, the potential increase follows the order: red > orange > yellow > green > blue. For the monoprotonated molecule (ER), it can be seen that deep red color is mainly located over the carbonyl group (C=O) and over two nitrogen atoms, which suggests that these moieties interact with the metallic surface through electrostatic forces. Meanwhile, the remaining part of the molecule interacts with chemical forces (nucleophilic reactivity). Similar observation was derived for the protonated ER^+^ species.

The total energies, dipole moments (*μ*), the quantum chemical descriptors HOMO and LUMO energies (*E*_HOMO_ and *E*_LUMO_), energy gap (Δ*E*), electrophilicity (*ω*), electronegativity (*χ*), the fraction of electron transferred (Δ*N*), the initial molecule–metal interaction energy (Δ*ψ*), and the back-donation energy (Δ*E*_b–d_), in both gas phase and in solution for the neutral ER molecule and its protonated form, ER^+^, are reported in Table SI 5.† Higher value of *E*_HOMO_, lower value of *E*_LUMO_ and lower value of Δ*E* are consistent with high metal–inhibitor interactions and therefore high protection efficiency, as stated previously.^61,62^ More importantly, the energy gap plays a significant role in the comparison among inhibitor molecules, especially when they have similar molecular structures. The molecule associated with lower Δ*E* value is also related to high softness, lower hardness and high chemical reactivity, and thereby high protection ability.^62,63^ From Table SI 5,† it can be seen that the inhibitor and its protonated form possesses higher *E*_HOMO_ value in solution. If the value of Δ*N* is more than zero, then the inhibitor molecule has the ability to move its electrons to the metal, and *vice versa*.^62,64,65^ The positive values of Δ*N* (Table SI 5†) for the neutral ER and for the protonated form of ER indicate that ER has the ability to transfer its electrons into empty d-orbitals of the surface iron atoms.

#### 3.7.2. Fukui functions

From our quantum chemical results discussed above, one could conclude that the ER molecule and its protonated form have a higher sensibility towards accepting and donating electrons from/to iron atoms. Therefore, in order to support the experimentally based hypothesis, it will be very useful to identify the most reactive sites.

In order to achieve this particular aim, the condensed Fukui functions of the inhibitor are considered as a valuable indices in estimating the local reactivity of the molecules.^66^ From the Fukui function, very useful data about the atomic site responsible for the electrophilic and nucleophilic reactions are derived.^62,67^ Fig. SI 12† presents the graphical representation of the most active sites, as well as the schematic representations of the tested inhibitor and its protonated form, with the number of atoms. For neutral ER molecule, the maximum positive magnitudes of Fukui functions are located on C7, C4, O1, C1 and N1. On the other hand, maximum negative magnitudes of Fukui functions are on N2, C3, and O1. In the protonated form (ER^+^), the maximum positive magnitudes of Fukui functions are located on C4, C8, C1, O1 and C6, while the maximum negative magnitudes of Fukui functions are located on N2, O4, C3, and N1.

### 3.8. Molecular dynamics simulation

**Fig. 8:**
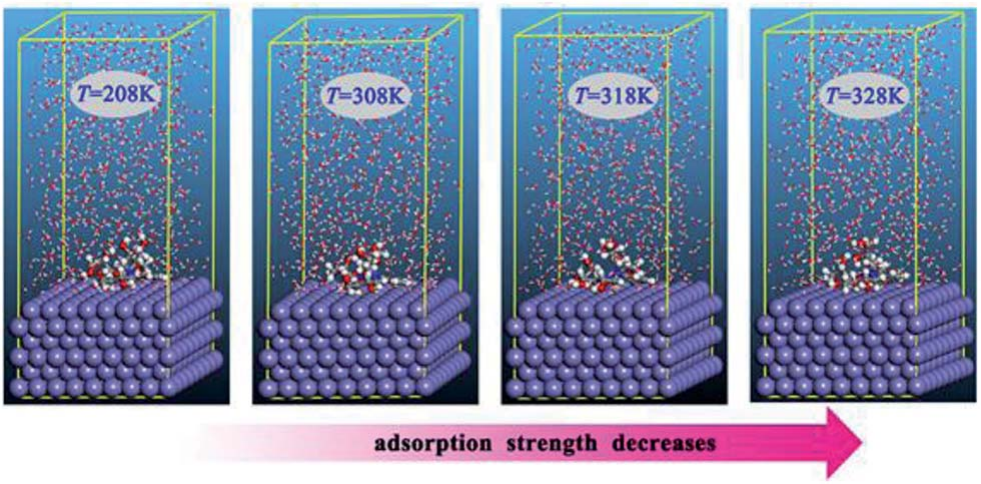
Equilibrium adsorption configurations of the inhibitor compound studied on Fe(110) surfaces with different simulated temperatures.

It has been well established that epoxides in ER have a great tendency to undergo ring-opening reactions in acidic solution. Thus, the open ring form of ER was used for the simulation. Fig. 8 depicts the equilibrium configurations of inhibitor molecules adsorbed on the surface of Fe(110) at different simulated temperatures. As presented in Fig. 8, it is probable that the inhibitor molecules are adsorbed on the Fe(110) surface through benzamide and hydroxyl groups.

The energy of adsorption in solution can be calculated following eqn (23):^68^

23 *E*_ads_ = *E*_total_ − (*E*_surf+water_ + *E*_ihn+water_) + *E*_water_

where all the symbols have their usual meanings.^62^ The adsorption energies in the present work were calculated from the average adsorption energy of the obtained equilibrium configurations. The obtained *E*_ads_ values are −534.2, −532.3, −529.5, and −513.7 kJ mol^−1^ when the simulated temperatures are 298 K, 308 K, 318 K, and 328 K, respectively. The results show that at all temperatures investigated, the adsorption energies are negative, and therefore, spontaneous adsorption can be expected. Generally, a more negative value of *E*_ads_ suggests stronger adsorption strength between an inhibitor molecule and the metal surface. Apparently, the absolute values of *E*_ads_ decrease with temperature increase, which is in agreement with the order of the observed inhibition efficiency.

## 4 Conclusions

In this work, we presented the synthesis of the epoxy monomer tetraglycidyl-1,2-aminobenzamide (ER). This epoxy monomer was characterized by ^1^H NMR and FT-IR spectroscopy. Based on the results obtained and presented, it can be concluded that:

- The viscosities of the studied solution showed a high dependence on temperature and concentration.

- ER solutions in ethanol showed a non-Newtonian fluid behavior, and their viscosities were found to depend on shear rate.

- The zero shear rate activation energy of ER/ethanol solutions varies between 0.60–11.90 kJ mol^−1^.

- The PDP results reveal that the epoxy monomer behaves as an anodic-type corrosion inhibitor.

- The EIS results suggest that ER protects the carbon steel from corrosion by the formation of a protective film at the metal surface.

- The values of standard free energy of adsorption indicate strong interaction between inhibitor molecules and carbon steel.

- DFT and molecular dynamics simulation parameters showed that ER molecules form strong bonds with the metallic surface. The theoretical results are consistent with experimental findings.

## 5 Author's contributions

O. Dagdag, A. El Harfi, and O. Cherkaoui carried out the experiments. Z. Safi and N. Wazzan carried out and discussed the DFT part. Lei Guo carried out and discussed the molecular dynamics simulation study. E. D. Akpan and Chandrabhan Verma assisted in collation of the results and carried out the computational calculations. E. E. Ebenso and Ramzi T. T. Jalgham conceptualised and designed the work and were part of the manuscript write-up.

## Conflicts of interest

There are no conflicts to declare.

## Supplementary Material

RA-009-C8RA09446B-s001
