# Supplementary material for: Rheological, electrochemical, surface, DFT and molecular dynamics simulation studies on the anticorrosive properties of new epoxy monomer compound for steel in 1 M HCl solution
Source: RSC Adv. 2019 Feb 5;9(8):4454–62. doi: 10.1039/c8ra09446b (PMC9060584; doi:10.1039/c8ra09446b)
Supplement: RA-009-C8RA09446B-s001 [file RA-009-C8RA09446B-s001.pdf]

Electronic Supplementary Material (ESI) for RSC Advances.
This journal is © The Royal Society of Chemistry 2019

Rheological, Electrochemical, Surface, DFT and Molecular Dynamics Simulation Studies on the Anticorrosive Properties of New Epoxy Monomer Compound for Steel in 1 M HCl Solution

Omar Dagdag[a], Ahmed El Harfi[a], Omar Cherkaoui[b], Zaki Safi[c], Nuha Wazzan[d], Lei Guo[e], E. D. Akpan[f,g], Chandrabhan Verma[f,g*], E. E. Ebenso[f,g*] and Ramzi T.T. Jalgham[h]

[a]*Laboratory of Agroresources, Polymers and Process Engineering (LAPPE), Department of Chemistry, Faculty of Science, Ibn Tofail University, BP 133, 14000 Kenitra, Morocco.*
[b]*Higher School of Textile and Clothing Industries, Laboratory REMTEX, BP 7731, Oulfa, Casablanca, Morocco.*
[c]*Al Azhar University-Gaza, Chemistry Department, Faculty of Science, P.O Box 1277, Gaza, Palestine.*
[d]*King Abdulaziz University, Chemistry Department, Faculty of Science, P.O Box 42805, Jeddah, 21589, Saudi Arabia.*
[e]*School of Materials and Chemical Engineering, Tongren University, Tongren, 554300, China.*
[f]*Department of Chemistry, Faculty of Natural and Agricultural Sciences, School of Chemical and Physical Sciences, North-West University, Private Bag X2046, Mmabatho 2735, South Africa.*
[g]*Material Science Innovation & Modelling (MaSIM) Research Focus Area, Faculty of Natural and Agricultural Sciences, North-West University, Private Bag X2046, Mmabatho 2735, South Africa*
[h]*Department of Oil and Gas, Faculty of Engineering, Bani Walid University, Bani Walid, Libya.*

Supplementary Information

ER: Brown viscous resin; ^{1}H-NMR (300 MHz, $CDCl_3$): δppm=2.38; 2.63 (dd, 2H, CH_2) (A,B), 2.77; 3.16 (m, 1H, CH oxirane) (X), 3.46 (dd, 2H, -N-CH_2) (C,D),3.61 (dd, 2H, -CO(N)-CH_2) (A,B), 6.94; 7.71 (s, 4H aromatic) (Ar); FTIR (cm^{-1}): 3270 (residual hydroxyl and amine groups), 2978, 2883 (C-H vibrations), 1585, 1500, 1450 (1,2- substituted aromatic ring), 1654 (C=O amide), 1396, 1095, 1043 (C-O), 930, 880 (oxirane ring).

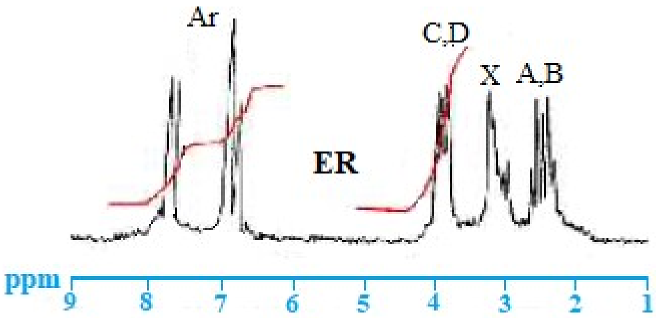

Fig SI 1 ^{1}H NMR spectra of ER.

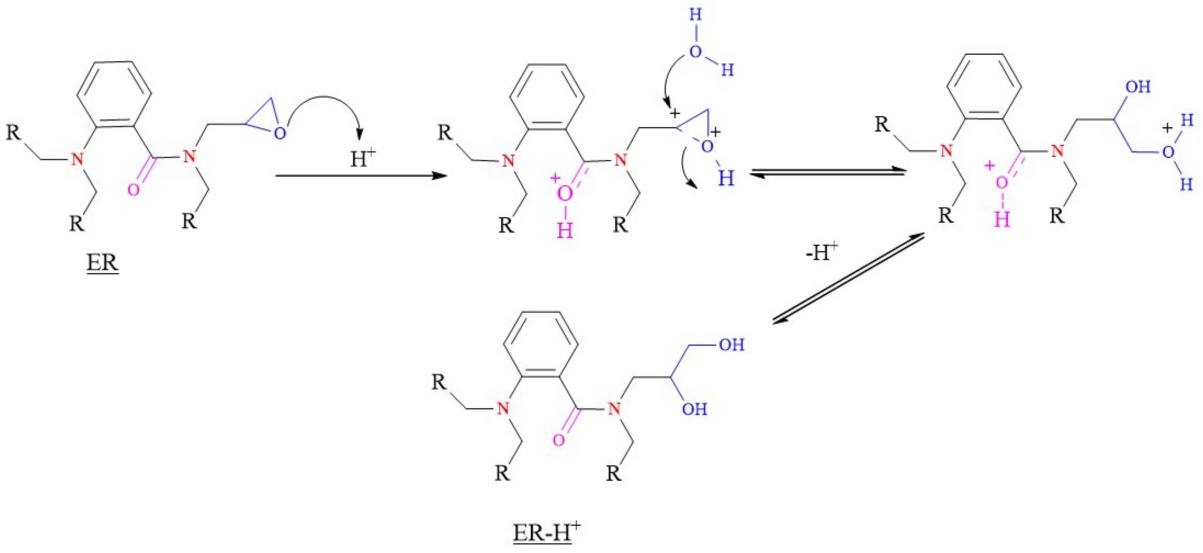

Fig. SI 2 Mechanism of ring opening reaction of epoxides of ER in acid solution.

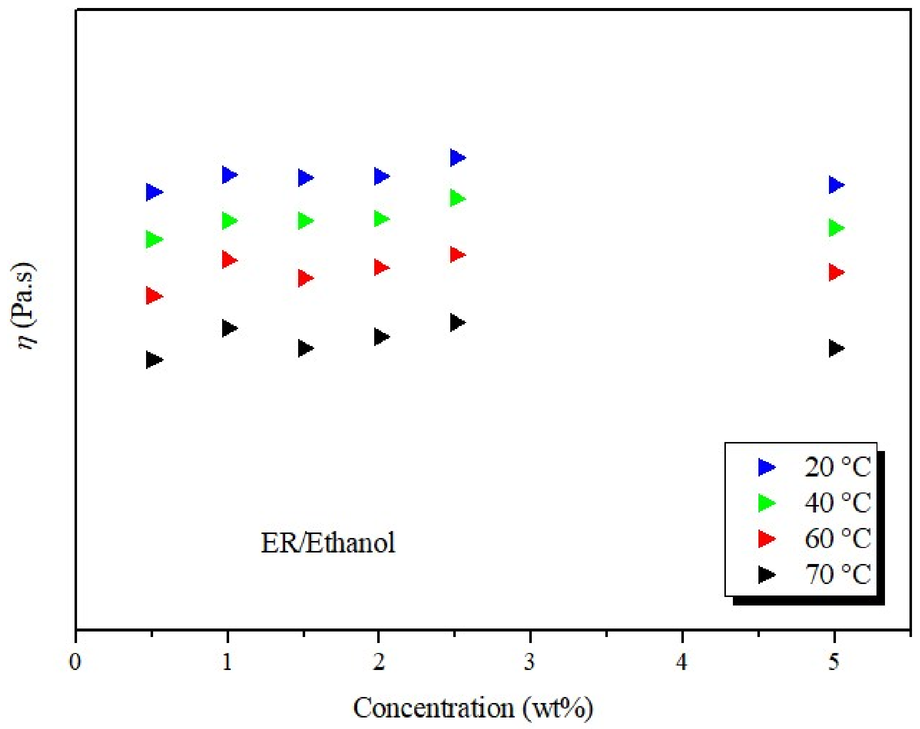

Fig. SI 3 The consequences of concentration on viscosity of ER/Ethanol at: 20, 40, 60 and 70 °C.

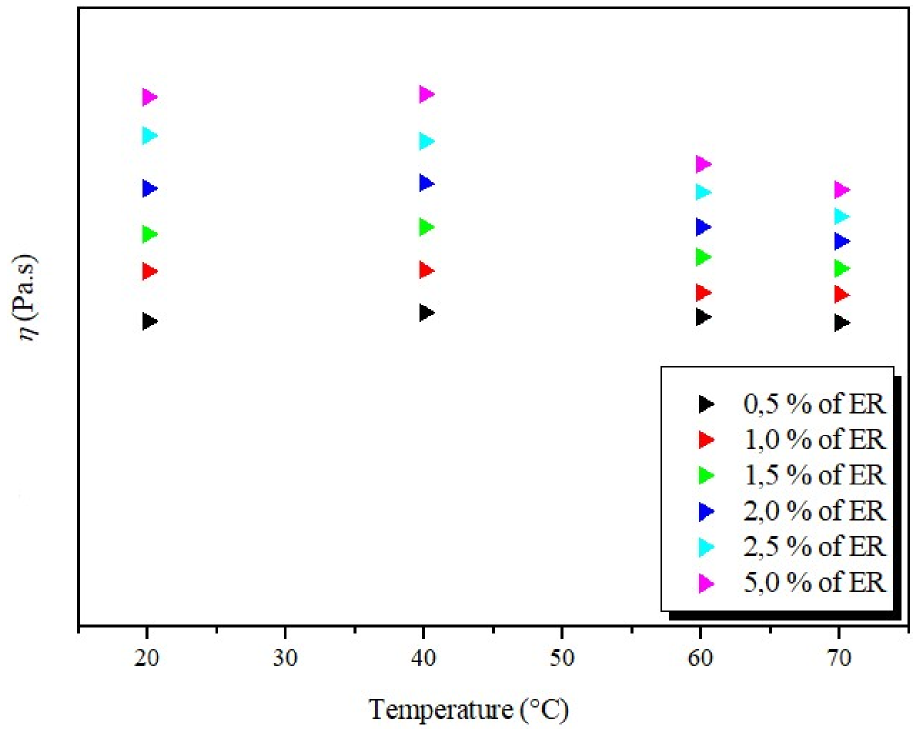

Fig. SI 4 Viscosity as a function of temperature of ER/Ethanol at various concentrations

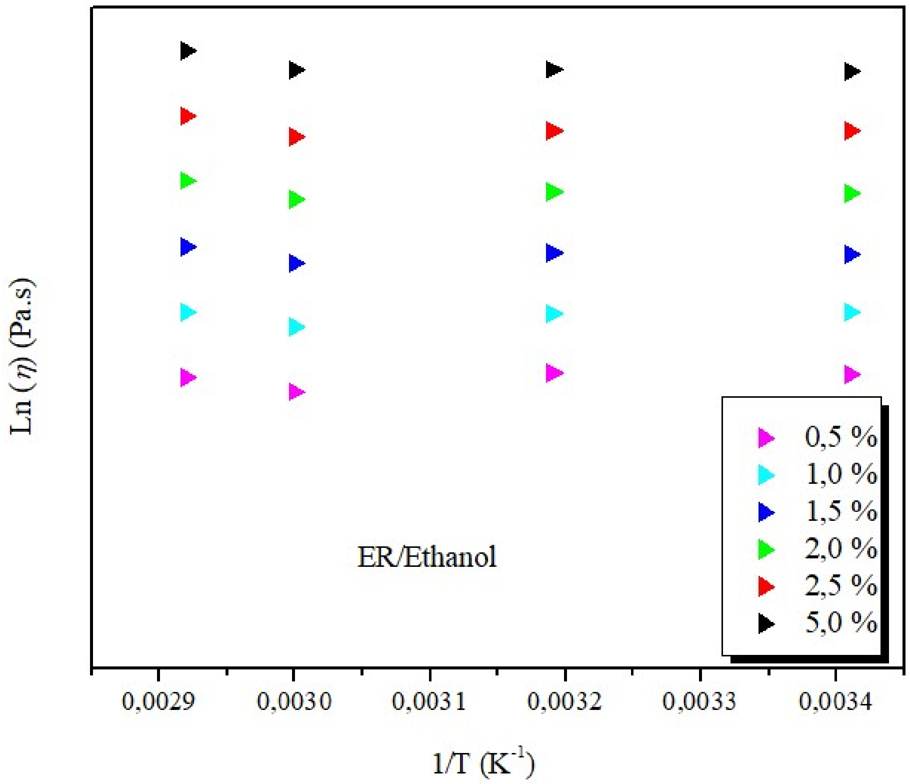

Fig. SI 5 Arrhenius plots for the zero shear viscosity of concentrations of ER/Ethanol.

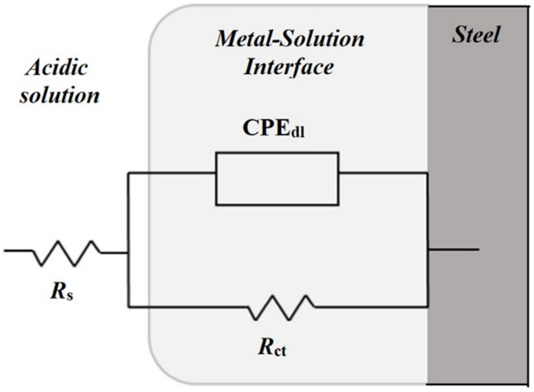

Fig. SI 6 Equivalent circuit used for the analysis of the EIS data.

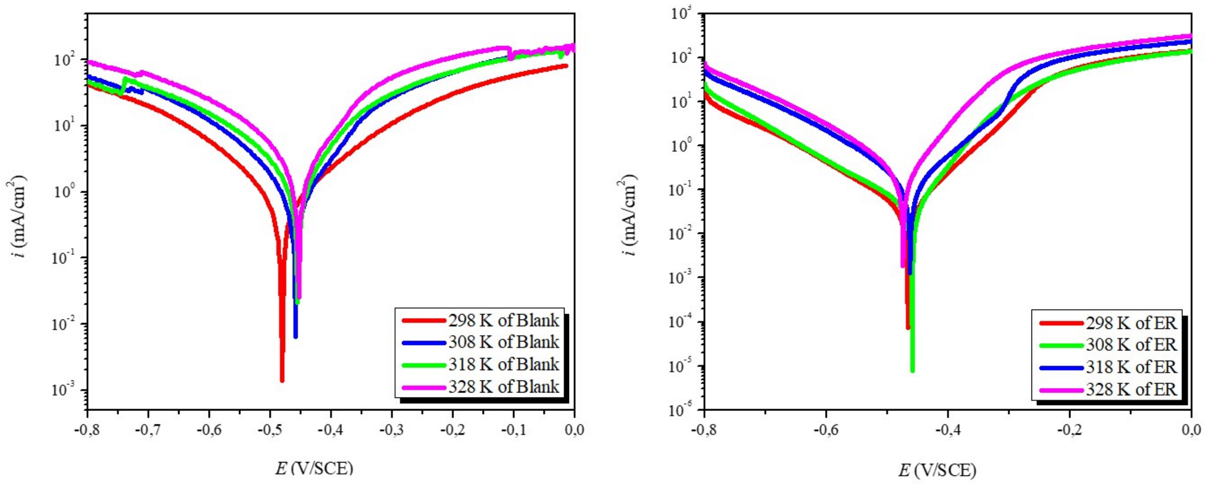

Fig. SI 7 Potentiodynamic polarization plots of carbon steel in 1 M HCl solution without and in the presence of 10^{-3} M of ER at varying temperatures.

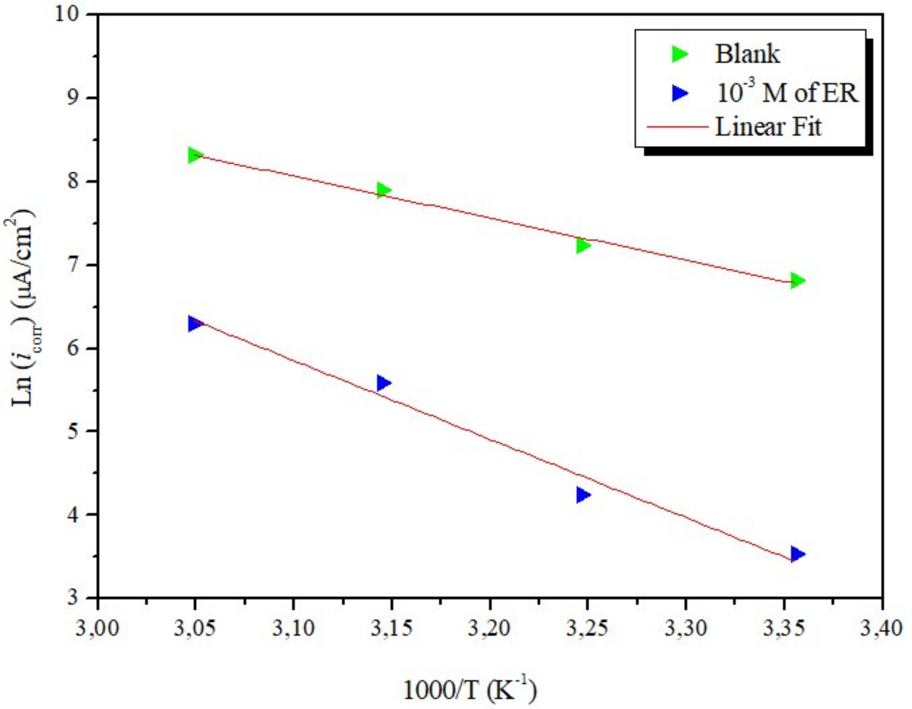

Fig. SI 8 The relationship between Ln (i_{corr})and 1/T for carbon steel in 1 M HCl solution without and in the presence of 10^{-3} M concentration of ER.

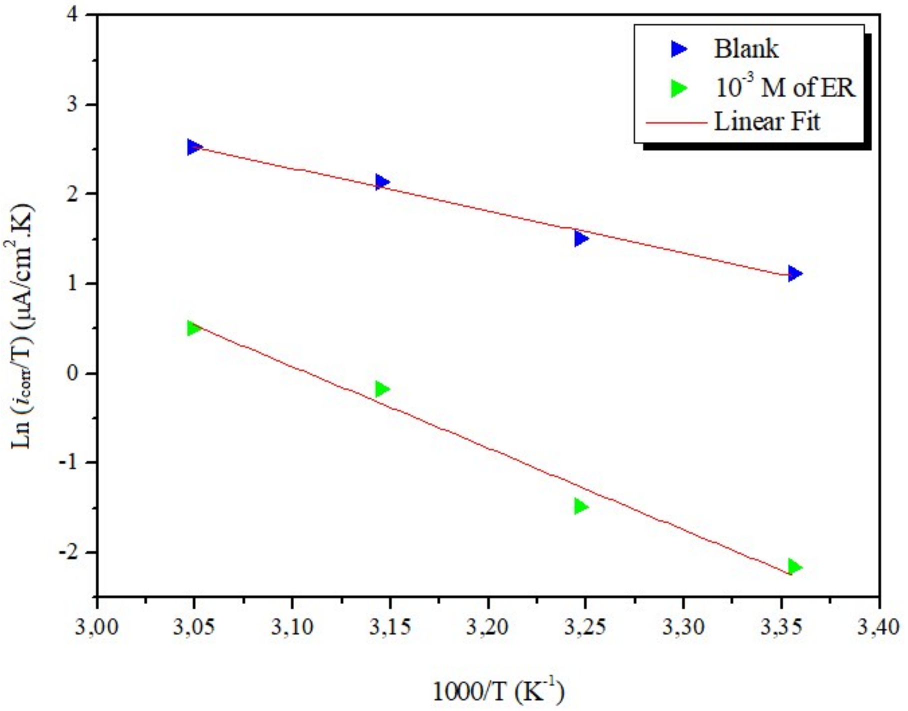

Fig. SI 9 Transition state plots for carbon steel in 1 M HCl solution without and in the presence of 10^{-3} M concentration of ER.

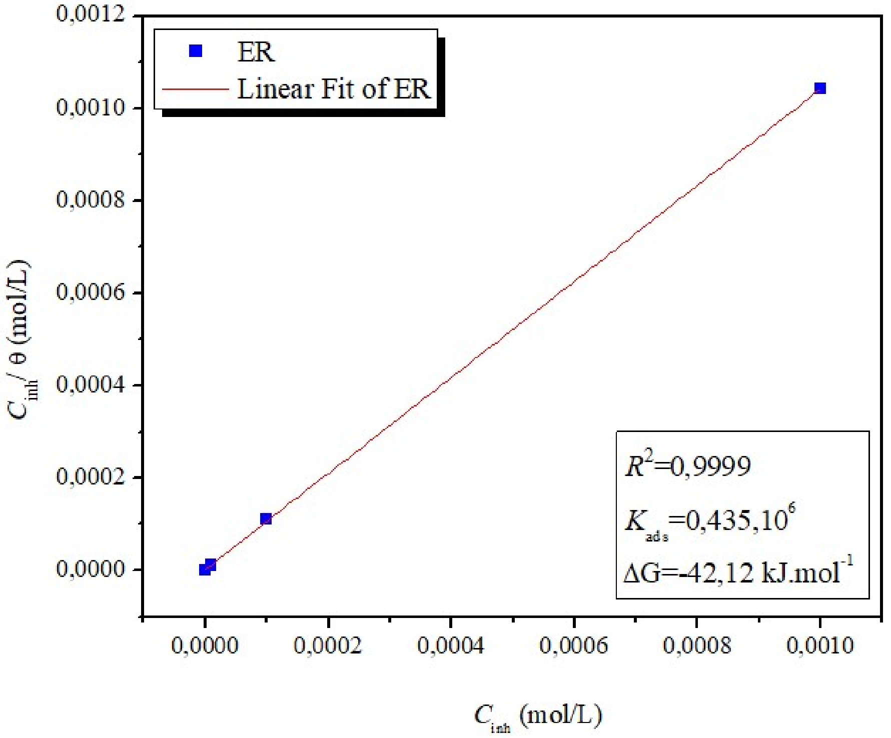

Fig. SI 10. Langmuir adsorption isotherm plot of ERon the carbon steel surface at 298 K.

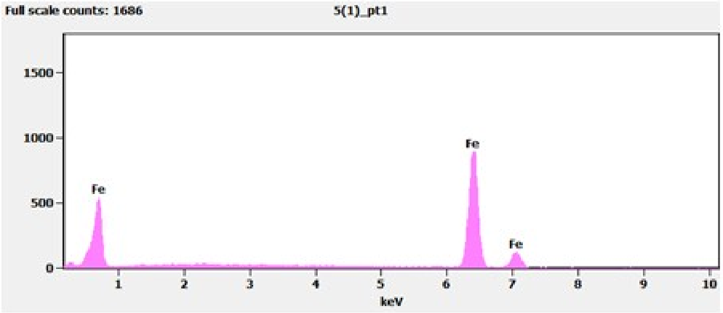

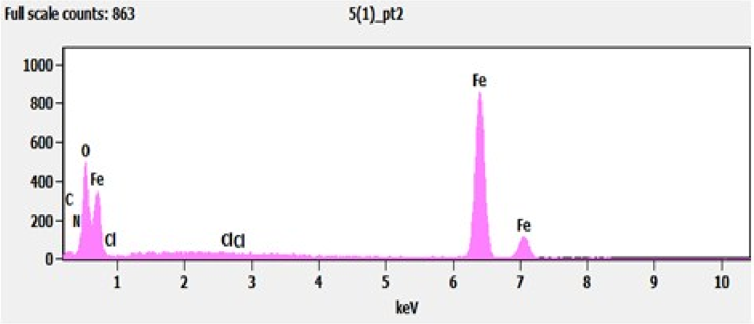

Fig. SI 11. EDX spectra of mild steel surface corroded in 1M HCl with and without ER.

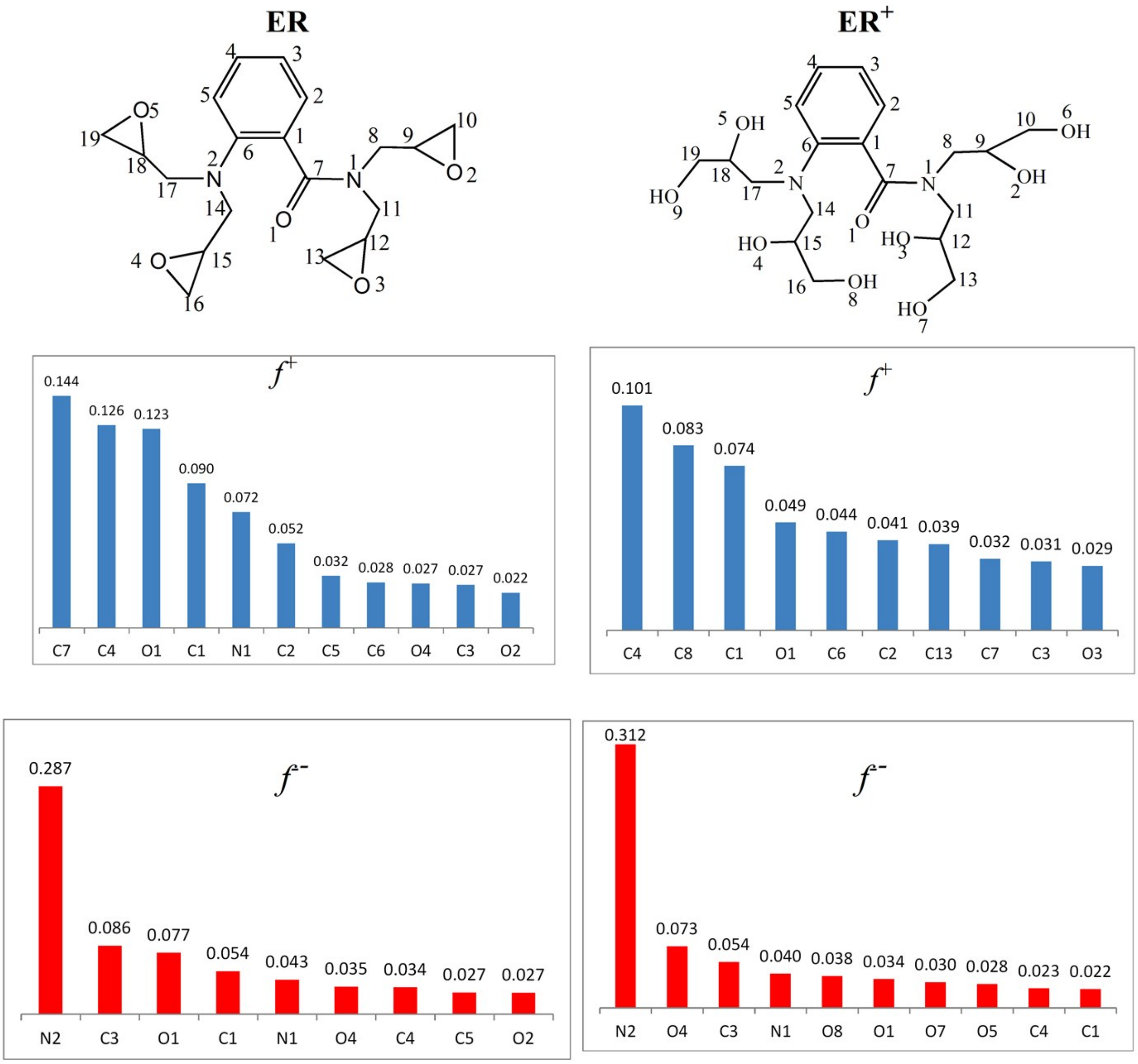

Fig. SI 12. Graphical presentation of the calculated Fukui indices of ER and its protonated form ER^{+}.

Table SI 1. Composition of the carbon steel.

C	Mn	Si	Al	Cr	Mo	Ni	Cu	Co	V	Fe
**0.11**	0.47	0.24	0.03	0.12	0.02	0.1	0.14	<0.001	<0.003	Balance

Table SI 2 Name, abbreviation, chemical structure and analytical data of the synthesized compound.

Inhibitor	Chemical structure	Analytical data
Tetraglycidyl-1,2-Aminobenzamide (ER)	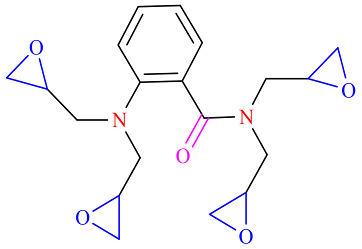	**ER:** Brown viscous resin, yield 92%; ^{1}H-NMR (DMSO-d_6, 300 MHz): δppm=2.38; 2.63 (dd, 2H, CH_2), 2.77; 3.16 (m, 1H, CH oxirane), 3.21; 3.46 (dd, 2H, -N-CH_2),3.21; 3.36: 3.61 (dd, 2H, -CO(N)-CH_2), 6.94; 7.71 (s, 4H aromatic); FTIR (cm^{-1}): 3270 (residual hydroxyl and amine groups), 2978, 2883 (C-H vibrations), 1585, 1500, 1450 (1,2-substituted aromatic ring), 1654 (C=O amide), 1396, 1095, 1043 (C-O), 930, 880 (oxirane ring).

Table SI 3: The impact of temperature on the electrochemical parameters for carbon steel in 1 M HCl and 10^{-3} M of ER.

T (K)	E_{corr} mV/SCE		i_{corr} μA/Cm^2		η%
	Blank	10^{-3} M of ER	Blank	10^{-3} M of ER	-
298	- 473	-464	916	34.31	96
308	- 459	-459	1390	69.70	95
318	- 455	-462	2700	268.85	90
328	- 453	-474	4100	546.82	87

	E_a (kJ/mol)	ΔH_a (kJ/mol)	ΔS_a (J.mol^{-1} K^{-1})
**Blank**	41.94	39.30	- 56.70
**10^{-3} M of ER**	78.40	75.90	38.07

Table SI 4 Activation parameters for carbon steel in 1 M HCl solution without and in the presence of ER.

Table SI 5 Calculated Quantum chemical parameters for epoxy compound and its protonated form obtained from DFT/B3LYB/6-311+G (d, p) in both gas phase and in solution.

	Gas		Solution	
	ER	ER^+	ER	ER^+
**Energy (a.u.)**	-1224.236344	-1530.223571	-1224.258717	-1530.253177
μ*	3.731	3.949	5.660	3.698
E_{HOMO} (eV)	-6.385	-6.615	-6.338	-6.511
E_{LUMO} (eV)	-0.968	-1.208	-0.881	-1.060
I	6.385	6.615	6.338	6.511
A	0.968	1.208	0.881	1.060
ΔE	5.417	5.407	5.457	5.451
χ	3.676	3.912	3.609	3.785
η	2.709	2.704	2.728	2.726
σ	0.369	0.370	0.367	0.367
ω	2.709	2.704	2.728	2.726
ΔN100	0.043	0.000	0.043	0.000
ΔN110	0.211	0.168	0.211	0.168
ΔN111	0.038	-0.006	0.038	-0.006
Δψ	1.020	0.882	1.054	0.948
ΔE_{b-d}	-0.677	-0.676	-0.682	-0.681
